# Disrupting ER-associated protein degradation suppresses the abscission defect of a weak *hae hsl2* mutant in Arabidopsis

**DOI:** 10.1093/jxb/erw313

**Published:** 2016-08-26

**Authors:** John Baer, Isaiah Taylor, John C. Walker

**Affiliations:** ^1^Division of Biological Science, University of Missouri, Columbia, MO 65211, USA; ^2^Interdisciplinary Plant Group, University of Missouri, Columbia, MO 65211, USA

**Keywords:** Abscission, *EBS3*, *EBS4*, ERAD, ER-associated degradation, genome sequencing, *HAESA*, *HAESA-LIKE 2*, suppressor.

## Abstract

In *Arabidopsis thaliana*, the process of abscission, or the shedding of unwanted organs, is mediated by two genes, *HAESA* (*HAE*) and *HAESA-LIKE 2* (*HSL2*), encoding receptor-like protein kinases (RLKs). The double loss-of-function mutant *hae-3 hsl2-3* is completely deficient in floral abscission, but, interestingly, the *hae-3 hsl2-9* mutant displays a less severe defect. This mutant was chosen for an ethyl methanesulfonate (EMS) screen to isolate enhancer and suppressor mutants, and two such suppressors are the focus of this study. Pooled DNA from the F_2_ generation of a parental backcross was analyzed by genome sequencing to reveal candidate genes, two of which complement the suppressor phenotype. These genes, *EMS-MUTAGENIZED BRI1 SUPPRESSOR 3* (*EBS3*) and *EBS4*, both encode mannosyltransferases involved in endoplasmic reticulum (ER)-associated degradation (ERAD) of proteins. Further analysis of these suppressor lines revealed that suppressor mutations are acting solely on the partially functional hsl2-9 mutant receptor to modify the abscission phenotype. Expressing a hsl2-9–yellow fluorescent protein (YFP) transgene in *ebs3* mutants yields a higher fluorescent signal than in *EBS3*/*ebs3,* suggesting that these mutants restore abscission by disrupting ERAD to allow accumulation of the hsl2-9 receptor, which probably escapes degradation to be trafficked to the plasma membrane to regain signaling.

## Highlight

Two suppressors of a weak abscission-deficient mutant were identified using genome sequencing. One suppressor was found to increase accumulation of the mutant hsl2-9 receptor compared with the parent genotype.

## Introduction

Abscission is a cell separation process that plants use to shed unwanted organs. This process has been best studied in the model plant *Arabidopsis thaliana*, which sheds its floral organs following pollination. Two receptor-like protein kinases, HAESA (HAE) and HAESA-LIKE 2 (HSL2), are required for abscission to occur, and the double loss-of-function mutant *hae hsl2* is completely deficient in abscission (Cho *et al.*, 2008; Stenvik *et al.*, 2008). The secreted peptide INFLORESCENCE DEFICIENT IN ABSCISSION (IDA) is the ligand of *HAE/HSL2*, and *ida* mutants also do not abscise (Butenko *et al.*, 2003, 2014; Meng *et al.*, 2016). Several proteins belonging to the *SOMATIC EMBRYOGENESIS RECEPTOR KINASE* (*SERK*) family have also been shown to function as co-receptors of *HAE/HSL2* (Meng *et al.*, 2016; Santiago *et al*., 2016)*. HAE/HSL2* signaling activates a mitogen-activated protein kinase (MAPK) cascade consisting of *MITOGEN-ACTIVATED PROTEIN KINASE KINASE 4* (*MKK4*) and *MKK5*, as well as *MITOGEN-ACTIVATED PROTEIN KINASE 3* (*MPK3*) and *MPK6* (Cho *et al.*, 2008). One target of MAPK phosphorylation is the transcription factor *AGAMOUS-like 15* (*AGL15*), which has been shown to bind to the promoter of *HAE* and regulate its transcription (Patharkar and Walker, 2015). Further, it has been shown that this signaling cascade constitutes part of a positive feedback loop to increase *HAE* expression just before abscission occurs (Patharkar and Walker, 2015). *HAE/HSL2* signaling has also been shown to increase expression of cell wall-modifying and hydrolytic enzymes in a region of cells at the base of flowers called the abscission zone (AZ; Niederhuth *et al.*, 2013*b*). The AZ consists of a layer of cells attaching organs that will be abscised to the plant, and separation of these cells, caused by the breakdown of the middle lamella by hydrolytic enzymes, allows floral organs to be shed (Niederhuth *et al.*, 2013*a*). Abscission of the floral organs in Arbidopsis occurs at approximately floral position 7–9, where position 1 is defined as the youngest flower after anthesis (Cho *et al.*, 2008). These positions correspond to floral developmental stage 16, which is defined by the withering and loss of petals, sepals, and stamen (Roeder and Yanofsky, 2006; Alvarez-Buylla *et al.*, 2010).

Here, we connect the process of abscission to the protein quality control and degradation systems present in the endoplasmic reticulum (ER). ER-mediated protein quality control (ERQC) and ER-associated degradation (ERAD) are mechanisms allowing correctly folded proteins to be exported while retaining misfolded proteins to attempt refolding, or eventually to dispose of terminally misfolded proteins to prevent accumulation (Liu and Li, 2014; Lannoo and Van Damme, 2015). To achieve this, lipid-linked glycan precursors are transferred onto nascent polypeptides to provide a substrate that can be easily modified by ERQC/ERAD machinery to move polypeptides through the folding process and either export or degrade them, depending on whether it achieves its native state (Liu and Li, 2014; Lannoo and Van Damme, 2015). To date, ERQC and ERAD processes have been extensively studied in yeast, but relatively little is known about how these processes function in plants. The leucine-rich repeat (LRR) receptor kinase BRI1 has been adopted as a model protein for ERQC/ERAD studies in Arabidopsis, and genetic screens looking for suppressors of weak *bri1* mutants have resulted in identifying several components of the ERQC and ERAD systems (Jin *et al.*, 2007, 2009; Hong *et al.*, 2009, 2012). Similarly, we demonstrate here that two mutants capable of suppressing the weak abscission defect of *hae-3 hsl2-9* were found to have mutations in the genes *EMS-MUTAGENIZED BRI1 SUPPRESSOR 3* (*EBS3*) and *EBS4*, both of which were shown to suppress the dwarf mutant *brassinosteroid-insensitive 1* (*bri1*) by disrupting ERAD (Hong *et al.*, 2009, 2012).

Genetic screens have identified a series of alleles for both *hae* and *hsl2* mutants (Niederhuth *et al.*, 2013*a*). Of importance here are the double mutants *hae-3 hsl2-3* (containing C222Y and G360R substitutions, respectively) and *hae-5 hsl2-4* (containing W522* and Q165* substitutions, respectively). Both of these double mutants display completely abscission-deficient phenotypes. Interestingly, the *hae-3 hsl2-9* mutant displays a weaker abscission defect (*hsl2-9* contains a P404L substitution), and was chosen for an ethyl methanesulfonate (EMS) screen because it could result in isolating both enhancer and suppressor mutants. The lesions of all relevant *hae/hsl2* mutant alleles are displayed in Supplementary Fig. S1A–E at *JXB* online. Through the course of this study, by identifying two mutants that suppress the weak abscission defect of *hae-3 hsl2-9*, we demonstrate that the HSL2 receptor is subject to the ERQC and ERAD systems. These two suppressor mutants further allowed us to elucidate that the hsl2-9 mutant receptor is biochemically functional and can signal to regain abscission in the suppressor mutants, illustrating that the ERQC and ERAD systems are often overly stringent in retaining and degrading misfolded proteins.

## Materials and methods

### Plant growth conditions

Plants were grown in a 16h light/8h dark cycle at temperatures of 20 °C during light and 18 °C during darkness, with 35–70% humidity. Columbia ecotype (Col-0) was used as the wild type. Mutations in the *ERECTA* (*ER*) and *GLABROUS* (*GL*) genes were introduced into several backgrounds throughout this study in order to identify contaminants easily.

### Generation of mutagenized seed for the genetic screen

Approximately 10 000 seeds of the *hae-3 hsl2-9 er gl* mutant were mutagenized with EMS according to the protocol as described by Weigel and Glazebrook (2006). An M_2_ population was grown and screened for individuals exhibiting enhancement or suppression of the weak abscission defect.

### Quantification of abscission phenotypes

Abscission phenotypes were quantified by counting the number of petals and sepals retained on each flower after treatment with a homemade brush device (Supplementary Fig. S2A). This device was constructed using two 7.5cm wide paintbrushes (bristles ~3.5cm long) with a glass rod running through holes in the paintbrush handles. Brushes were spaced 8cm apart along the glass rod using tape to ensure only the bristles of the brush would touch at the bottom. Two 1cm spacers were taped to one brush to provide a space between brushes where inflorescences rest during treatment and prevent brushes from squashing or damaging the plant. The device essentially hinges open to allow placement of inflorescence between the brushes, then is closed so the bristles brush the entire inflorescence as the device is pulled upward (Supplementary Fig. S2B). This is then repeated once after rotating the device ~90° around the inflorescence (rotate the device ~90° when looking down at the top of the inflorescence) to ensure every flower was treated (Supplementary Fig. S2C). The number of petals and sepals still attached was recorded for every flower from position 1 to 15. The average number of petals and sepals retained for positions 8–15 were then summed. The rationale behind this is that abscission has occurred fully by position 8, and after this point very few additional floral organs will abscise. Summing the average number of petals and sepals retained also allows for straightforward statistical analysis using pairwise *t*-tests assuming unequal variance to determine if significant differences between phenotypes are present, and *P*-values were determined by Bonferonni correction (Dunn, 2012).

### Genome sequencing

The two suppressor lines (lines 1.6 and 3.3) to be analyzed by genome sequencing were backcrossed to the *hae-3 hsl2-9 er gl* parent, and a segregating F_2_ population was grown. DNA was extracted using a DNeasy Plant Mini Kit (Qiagen, Cat. No. 69104) from pooled leaf tissue from 30 suppressed individuals for both suppressor lines, and also 30 non-suppressed individuals from the F_2_ of the line 1.6 backcross. DNA was sequenced on Illumina HiSeq 2500 and reads were aligned to the TAIR10 genome using Bowtie2 alignment software (Langmead and Salzberg, 2012). The resulting sequence alignment file was further analyzed using samtools and bcftools to create a list of variants between sequencing reads and the reference genome (Li *et al.*, 2009). For more information on bioinformatics work flow, see Supplementary File S1. The list of variants was then refined by selecting only variants containing a base pair change from guanine to adenine or from cytosine to thymine, as these are the most common mutations caused by EMS in Arabidopsis. The number of reads containing each variant [i.e. single nucleotide polymorphism (SNP)] was then divided by the total number of reads aligning to that position to give the proportion of reads containing each SNP. These proportions were then plotted by chromosomal position to allow identification of a ‘linked region’ with a higher overall proportion of reads containing SNPs in the two suppressed pools when compared with non-suppressed pools, which would be expected to contain the causal mutation. This method of genome sequencing by parental backcross to identify EMS-generated SNPs was previously described by Allen *et al.* (2013).

### Cloning

Constructs containing genomic fragments of *EBS3* and *EBS4* were obtained from the laboratory of Jianming Li, University of Michigan, and were transformed into the *hae-3 hsl2-9 ebs3 er gl* and *hae-3 hsl2-9 ebs4 er gl* mutants, respectively, by floral dip (Clough and Bent, 1998). T_1_s were selected on half-stength Murashige and Skoog (1/2 MS) plates (2.16g of MS basal medium per 1 liter, supplemented with 1% sucrose, w/v) containing 0.8% agar and kanamycin (K50), and then transplanted onto soil following selection. The *HAEpr:HAE-YFP* construct used was previously described (Taylor *et al.*, 2016). This construct was then mutagenized to introduce the C222Y mutation of *hae-3* by the Quick-Change site-directed mutagenesis method (Agilent Technologies, Santa Clara, CA, USA). To create HSL2–yellow fluorescent protein (YFP), a 6068bp fragment containing the entire *HSL2* gene and 3002bp upstream promoter region was cloned into the *Not*I site of pE6c (Dubin *et al.*, 2008). This construct was then mutagenized with primers to create the P404L substitution of *hsl2-9* by the Quick-Change site-directed mutagenesis method (Agilent Technologies). Both the *hae-3-YFP* and *hsl2-9-YFP* constructs were then transferred to pGWB601 by Gateway recombination (Nakamura *et al.*, 2010; Gateway LR Clonase II Enzyme Mix from Invitrogen Cat. No. 11791020), and then transformed separately into *hae-3 hsl2-9* by floral dip (Clough and Bent, 1998). T_1_s were then selected with Basta.

### Endo H assay

Tissue was frozen and ground in microcentrifuge tubes with pestles for each genotype. For *hae-3 hsl2-3 HSL2-YFP*, *hae-3 hsl2-9 er gl hae-3-YFP ebs3*, and *hae-3 hsl2-9 er gl hsl2-9-YFP ebs3* plants, two whole flowers (stage 16 and late stage 16) were ground and resuspended in 30 µl of SDS sample buffer. All samples were then boiled for ~3min and centrifuged to pellet tissue debris. Half of the supernatant of each sample was then treated with 1000U of Endo Hf with 1× Glyco Buffer 3 (NEB Cat. No. P0703S) for 1h at 37 °C (according to the manufacturer’s recommendations). Half of each sample was also left untreated. Samples were then separated by SDS–PAGE and immunoblotted as described below.

### Fluorescence microscopy and image quantification

Fluorescent images of stage 16 and late stage 16 flowers were obtained using a Canon EO5 6D camera in conjunction with a Zeiss Discovery.V12 fluorescent stereoscope. Images were then processed using Adobe Photoshop, and the fluorescent signal was quantified using ImageJ software (Schneider *et al.*, 2012). This allowed highlighted pixels, or those giving off fluorescent signal, to be counted within an area defined by a border traced around AZs. These values were then corrected by subtracting the average of three background readings taken from the same photo, and then normalized to untransformed wild type. Statistical significance was determined by Student’s *t*-test, with a *P*-value <0.05.

### Analysis of YFP fusion proteins by western blot

Two whole flowers (stage 16 and late stage 16) were frozen and ground in microcentrifuge tubes with pestles for each genotype (untransformed wild-type, *hae-3 hsl2-9 hsl2-9-YFP er gl ebs3*, and *hae-3 hsl2-9 hsl2-9-YFP er gl EBS3/ebs3*), resuspended in 30 µl of SDS sample buffer, and boiled for ~3min. A 10 µl aliquot of each sample was separated by SDS–PAGE on an 8% acrylamide gel, blotted to a nitrocellulose membrane, stained with Ponceau-S, and imaged. Blots were then blocked with 4% BSA in phosphate-buffered saline with 0.1% Tween-20 (PBS-T) for 1h, probed with anti-green fluorescent protein (GFP) antibody overnight at 4 °C, rinsed with PBS-T four times for 5min each, incubated with anti-rabbit–horseradish peroxidase (HRP) (1:2500 dilution in 1% BSA in PBS-T, Cell Signaling Technologies, Danvers, MA, USA) for 1h at room temperature, rinsed again with PBS-T four times for 5min each, incubated with chemiluminescent substrate (Super Signal West Pico, Life Technologies, Carlsbad, CA, USA), and imaged using BioRad Chemidoc.

### RNA isolation and cDNA synthesis

Leaf RNA was isolated using TRIzol reagent (Ambion), each sample was split, and 2 µg of RNA was treated with DNase (Thermo Scientific) while 2 µg of RNA was left untreated. cDNA was then synthesized from RNA (both DNase treated and untreated) using SuperScript III (Invitrogen) and used in PCR (additional reactions were run with cDNA reactions lacking reverse transcriptase as a control) with primers flanking introns containing SNPs (as mapped by genome sequencing). For primer sequences and descriptions, see Supplementary File S3.

## Results

### Isolation of enhancers and suppressors of *hae-3 hsl2-9*

**Fig. 1.**
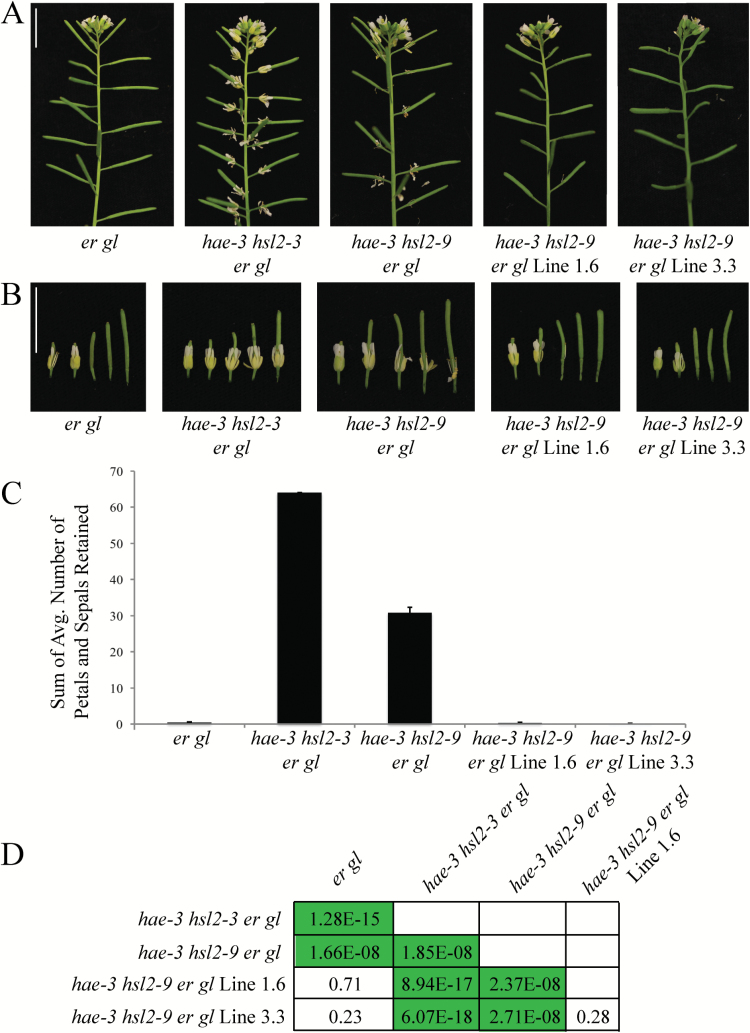
Mutant lines 1.6 and 3.3 suppress the abscission defect of *hae-3 hsl2-9 er gl*. (A) Photos of entire inflorescences showing abscission phenotypes of *er gl*, *hae-3 hsl2-3 er gl*, *hae-3 hsl2-9 er gl*, *hae-3 hsl2-9 er gl* Line 1.6, and *hae-3 hsl2-9 er gl* Line 3.3. (B) Stage 15 and the next four older flowers from the same inflorescences. (C) Quantification of abscission phenotypes. The number of petals and sepals retained was counted for floral positions 1–15 and averaged across 10 individuals. Averages of positions 8–15 were then summed for each genotype (SD error bars shown). (D) Table of *P*-values from statistical analysis using Student’s *t*-test. Values of statistical significance following Bonferroni correction (*P*<0.005) are highlighted. (This figure is available in colour at *JXB* online.)

Seed from the *hae-3 hsl2-9 er gl* mutant was mutagenized with EMS to screen for enhancers and suppressors of the abscission-deficient phenotype. The *hae-3 hsl2-9* mutant was chosen due to its weaker abscission deficiency, as compared with *hae-3 hsl2-3*, allowing for isolation of both enhancers and suppressors. Approximately 10 000 M_1_ seeds were grown, and progeny were harvested in 24 pools. Approximately 900 seeds were grown from each pool (~21 600 total plants) and screened in the M_2_ generation, resulting in isolation of 50 enhancers and 24 suppressors. These lines were scored based on the strength of their abscission phenotype, and those with the strongest abscission defect or rescue were regrown to confirm their phenotype in the M_3_ generation. Several enhancer and suppressor lines were backcrossed to the *hae-3 hsl2-9 er gl* parent to obtain a segregating F_2_ generation. Secondary mutations were found in the *HSL2* gene of three backcrossed enhancer lines when Sanger sequenced in the F_2_ generation. From this, we inferred that the majority of the enhancer lines were probably *HSL2* second site mutations. This prompted us to shift focus primarily to the suppressor lines, three of which were backcrossed as well. Of these three lines (named lines 1.6, 3.3, and 8.7), only lines 1.6 and 3.3 displayed the suppressor phenotype in the F_2_ generation (closely resembling wild-type abscission), and these lines became the focus of this study (Fig. 1A, B).

The phenotypes of suppressor lines 1.6 and 3.3 were quantified by counting the number of petals and sepals retained at each floral position, from position 1 to 15, after treatment with a homemade brush device (Supplementary Fig. S2A; see the Materials and methods). Given that pre-abscission flowers have four petals and four sepals, the maximum number that could be retained on one flower post-abscission is eight. The wild type and *er gl* retain none of their floral organs post-abscission (after stage 16), while the *hae-3 hsl2-3 er gl* mutant retains all of its floral organs. These genotypes represent the low and high end of the range of abscission phenotypes, with *hae-3 hsl2-9 er gl* falling between them (Supplementary Fig. S1F). The number of petals and sepals retained at each floral position was averaged for 10 individuals of each genotype, and the averages for positions 8–15 were summed. This gives a good overall quantitative description of the total number of floral organs retained post-abscission, and clearly shows the differences between phenotypes (Fig. 1C). The average number of petals and sepals retained for individual floral positions is displayed in Supplementary Fig. S1G. Statistically significant differences between phenotypes were determined by pairwise *t*-tests assuming unequal variance (*P*-values determined by Bonferroni correction are displayed in Fig. 1D). Importantly, there are clear statistically significant differences between the *hae-3 hsl2-9 er gl* parent and the two suppressor lines.

### Genome sequencing of lines 1.6 and 3.3 by parental backcross

Genome sequencing was employed to map EMS-generated SNPs and generate a list of candidate genes probably causing the suppressor phenotype. This was accomplished by crossing each suppressor line back to the parental *hae-3 hsl2-9 er gl* mutant. The F_2_ generation of these crosses was observed to segregate at an ~1:3 ratio of suppressed to non-suppressed individuals, suggesting a single recessive causal mutation. To identify the causal mutations, DNA was isolated from two pools of tissue (suppressed and non-suppressed individuals) from the F_2_ of the line 1.6 backcross. We chose to sequence a pool of non-suppressed 1.6 DNA in order to identify non-causal mutations that might have been present in the parental genome prior to mutagenesis. We also sequenced a single pool of tissue (suppressed individuals) from the F_2_ of the line 3.3 backcross.

**Fig. 2.**
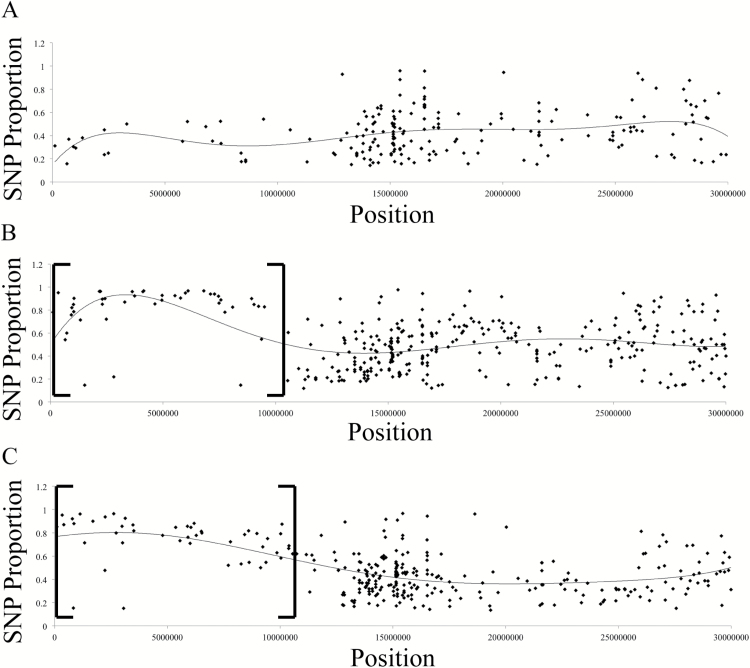
Genome sequencing of suppressor lines 1.6 and 3.3. Proportion of reads containing SNPs on chromosome 1 for (A) pool of non-suppressed individuals from a line 1.6 backcross, (B) pool of suppressed individuals from a line 1.6 backcross, and (C) pool of suppressed individuals from a line 3.3 backcross. Brackets indicate the region on chromosome 1 with a higher overall SNP Proportion that is linked to the suppressor phenotype.

Reads were aligned to the TAIR10 reference genome using Bowtie2 alignment software, and alignment files were further analyzed using samtools and bcftools to generate a list of variants between the sequenced genome and the TAIR10 reference genome; for work flow and commands used, see Supplementary File S1 (Li *et al.*, 2009; Langmead and Salzberg, 2012). For each variant, the number of reads containing the alternative called-base (SNP) was divided by the total number of reads aligning to that position to give the proportion of reads containing the SNP. This number was termed ‘SNP Proportion’, and means that if every read contained an alternative called-base at one position, then the SNP Proportion would be 1. SNP Proportion was plotted for every variant by chromosomal position to reveal a linked region on chromosome 1 for both suppressor pools containing variants with a higher SNP Proportion than in the non-suppressed pool (Fig. 2A–C). Graphs for chromosomes 1–5 are presented in Supplementary Fig. S3. SNPs in this region are linked to the causal mutation and are present in a higher proportion of reads due to the selection for the suppressor phenotype when pooling F_2_ tissue. We expected the SNP Proportion to increase closer to the causal mutation, with the SNP causing the suppressor phenotype to have an SNP Proportion of 1; however, we observed in the linked regions that the SNP proportion only approached 0.9. This is probably due to incorrectly scoring suppressed F_2_ individuals when pooling tissue. There are sometimes subtle differences between the suppressed phenotypes of lines 1.6 or 3.3 and the partial abscission defect of the *hae-3 hsl2-9 er gl* parent, and it is not unreasonable to think that one or two individuals included in the suppressed pools were not homozygous for the causative mutations. This may be due to variation in abscission that occurs due to other unknown endogenous factors, or these mutations may exhibit weak semi-dominance with incomplete penetrance.

The linked regions for line 1.6 and line 3.3 allowed us to narrow the number of variants present in the whole genome down to a reasonable number, with 46 variants present at an SNP Proportion >0.85 in line 1.6 and 37 variants in line 3.3. This number was further reduced by selecting only non-synonymous variants in genic regions and mutations at exon–intron junctions, leaving only 16 candidates in line 1.6 and 13 candidates in line 3.3 (all candidate genes are listed in Supplementary File S2). Searching the Arabidopsis genome by the chromosomal position of these variants allowed us to determine the genes containing these SNPs. From here, we noticed the candidate genes *EMS-MUTAGENIZED BRI1 SUPPRESSOR 3* (*EBS3*) and *EMS-MUTAGENIZED BRI1 SUPPRESSOR 4* (*EBS4*) in line 1.6 and line 3.3, respectively, and these genes, both being mannosyltransferases involved in the same biochemical pathway, became the most likely candidates. Further investigation revealed that these genes have both been previously described to suppress the semi-dwarf phenotype of weak *BRASSINOSTEROID-INSENSITIVE 1* (*bri1*) mutants (Hong *et al.*, 2009, 2012). The mannosyltransferase activity of both *EBS3* and *EBS4* is involved in the assembly of *N*-glycans in the ER, which are heavily utilized in the ERQC and ERAD systems of proteins.

Interestingly, the SNPs present in both of these genes are located in splice sites of introns. PCR using cDNA derived from the mRNA of line 1.6 leaf tissue, as well as primers flanking the intron containing the SNP, results in a larger product than that amplified from cDNA of the *hae-3 hsl2-9 er gl* mutant (Supplementary Fig. S4A). This suggests that this mutation interferes with splicing and results in retention of the intron containing the SNP (Supplementary Fig. S4C). Repeating this experiment for line 3.3 results in a slightly smaller product from line 3.3 compared with the *hae-3 hsl2-9 er gl* mutant (Supplementary Fig. S4B). Sequencing this product reveals that the mutated intron, as well as 22bp of the following exon, is not contained in the product, and suggests activation of a cryptic splice site downstream of the mutation (Supplementary Fig. S4D). In both cases, the coding region would be shifted out of frame, resulting in premature stop codons.

### *gEBS3* and *gEBS4* transgenes complement lines 1.6 and 3.3, respectively

**Fig. 3.**
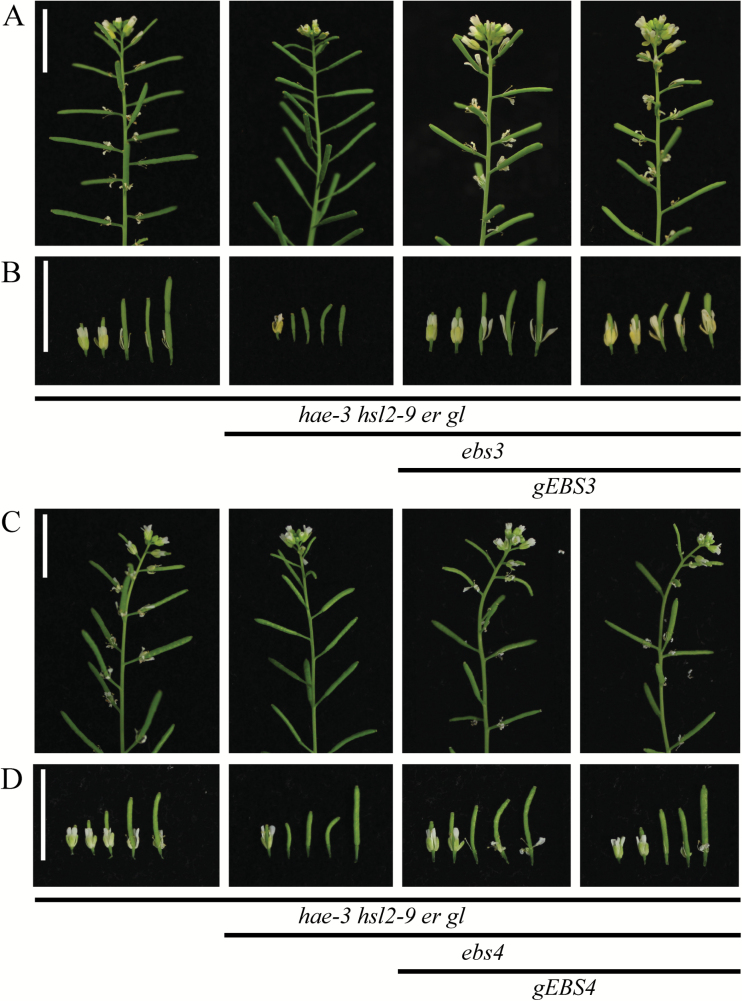
*gEBS3* and *gEBS4* transgenes complement suppressor lines 1.6 and 3.3, respectively. (A) Photos of entire inflorescences of *hae-3 hsl2-9 er gl*, *hae-3 hsl2-9 er gl ebs3*, *hae-3 hsl2-9 er gl ebs3 gEBS3* Line 6, and *hae-3 hsl2-9 er gl ebs3 gEBS3* Line 10. (B) Stage 15 and the next four older flowers from the same inflorescences. (C) Photos of entire inflorescences of *hae-3 hsl2-9 er gl*, *hae-3 hsl2-9 er gl ebs4*, *hae-3 hsl2-9 er gl ebs4 gEBS4* Line 1, and *hae-3 hsl2-9 er gl ebs4 gEBS4* Line 2. (D) Stage 15 and the next four older flowers from the same inflorescences. (This figure is available in colour at *JXB* online.)

To confirm that the suppression of the abscission-deficient phenotype in line 1.6 is caused by the *ebs3* mutation, a genomic version was transformed into the suppressor line 1.6. Several independent T_1_ lines were generated (Fig. 3A, B) and clearly show an abscission defect. Similarly, a genomic version of the *EBS4* gene was transformed into suppressor line 3.3. Independent T_1_ lines were again observed to have an abscission defect similar to the *hae-3 hsl2-9 er gl* parent (Fig. 3C, D), suggesting that the mutation in *ebs4* in line 3.3 is causing the suppression of the abscission-deficient phenotype. Abscission phenotypes for complemented lines were quantified as described above and the average number of petals and sepals retained for individual floral position is displayed in Supplementary Fig. S5A, B. The sum of the average number of petals and sepals retained for positions 8–15 is also shown (Supplementary Fig. S5C, D), with statistically significant differences present between both suppressors and complemented lines (determined by *t*-test assuming unequal variance, *P*-value <0.05).

### Identifying which receptor acts to regain abscission

The assembly of *N*-glycans is critical for the proper function of ERQC and ERAD (Lannoo and Van Damme, 2015). *N*-Glycans are transferred onto nascent polypeptides in the lumen of the ER, and are recognized by the protein folding machinery, quality control systems, and ultimately the signal for ERAD if proteins never achieve proper folding. This is probably the case with the hae-3 and hsl2-9 mutant receptors in *hae-3 hsl2-9 er gl*, as their mutations would cause structural imperfections prohibiting proper folding and causing them to be degraded. The *hae-3 hsl2-9 ebs3 er gl* and *hae-3 hsl2-9 ebs4 er gl* mutants, however, would not assemble the necessary *N*-glycans, allowing hae-3 and/or hsl2-9 to escape degradation and be trafficked to the plasma membrane, as was previously reported to be the case with the *ebs3-1 bri1-9* and *ebs4 bri1-9* mutants (Hong *et al.*, 2009, 2012). If this were the case in the suppressor mutants, then one or both of the receptors would have to be biochemically functional to be able to bind ligand and signal to regain abscission. We hypothesized that the hsl2-9 receptor is probably functional due to the change in severity of the abscission defect between *hae-3 hsl2-3 er gl* and *hae-3 hsl2-9 er gl* (Fig. 1).

**Fig. 4.**
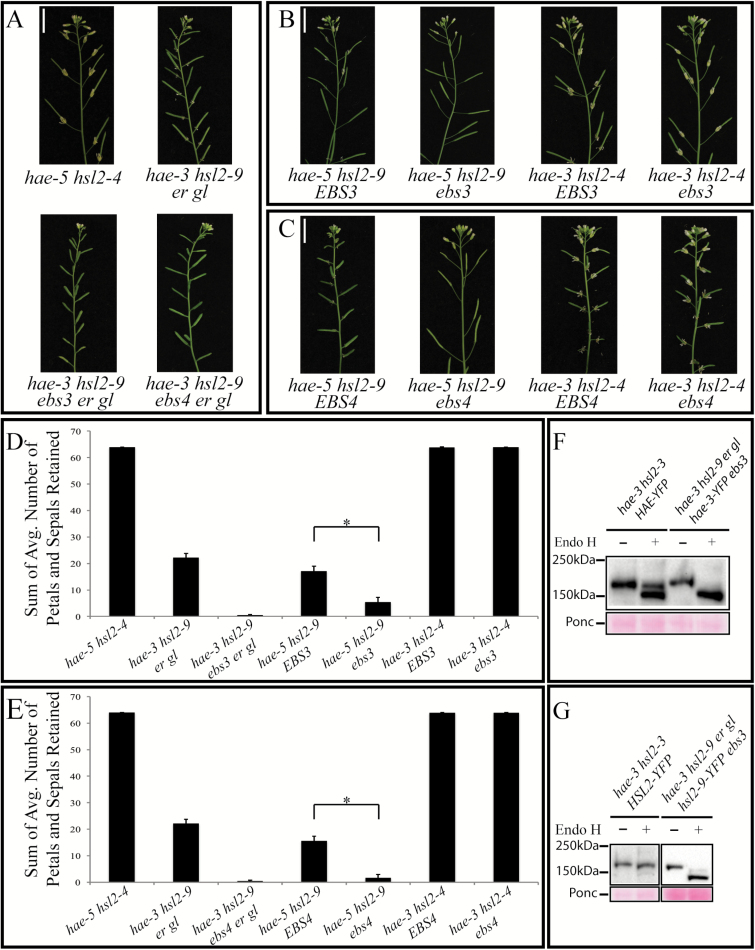
The hsl2-9 receptor is functioning to regain abscission. (A) Photos of entire inflorescences of *hae-5 hsl2-4* (both null alleles), *hae-3 hsl2-9 er gl*, *hae-3 hsl2-9 ebs3 er gl*, and *hae-3 hsl2-9 ebs4 er gl.* (B) Partial abscission defect of *hae-5 hsl2-9 EBS3*, no abscission defect of *hae-5 hsl2-9 ebs3*, and complete abscission defect of *hae-3 hsl2-4 EBS3* and *hae-3 hsl2-4 ebs3.* (C) Partial abscission defect of *hae-5 hsl2-9 EBS4*, no abscission defect of *hae-5 hsl2-9 ebs4*, and complete abscission defect of *hae-3 hsl2-4 EBS4* and *hae-3 hsl2-4 ebs4.* (D, E) Quantification of abscission phenotypes (by the same method as described above) for *ebs3* mutants (D) and *ebs4* mutants (E). Statistical significance determined by Student’s *t*-test (*P*-value <0.05, SD error bars shown). (F) Western blots showing sensitivity and mobility shift of HAE–YFP and hae-3–YFP receptors to Endo H digestion (blots probed with anti-GFP antibody). Ponceau stain of non-specific band for loading control. (G) Western blots showing the sensitivity of HSL2–YFP and hsl2-9–YFP receptors to Endo H digestion (blots probed with anti-GFP antibody). Ponceau stain of non-specific band for loading control. (This figure is available in colour at *JXB* online.)

To test this hypothesis, we crossed both suppressor mutants to the double null mutant *hae-5 hsl2-4*. The F_2_ generation of these crosses was genotyped looking for *hae-3* or *hsl2-9* paired with the alternative null receptor (i.e. *hae-3* paired with *hsl2-4* and *hae-5* paired with *hsl2-9*). Each of these pairs also needed either homozygous *EBS3* or *ebs3* (or *EBS4* and *ebs4*), meaning we needed four different triple homozygous mutants for each suppressor. All of these mutants were successfully genotyped in the F_2_ generation (Fig. 4A–C), and further analyzed in the F_3_ to quantify their abscission phenotypes (Fig. 4D, E). Again, the sum of the average number of petals and sepals for positions 8–15 is shown here (individual positions 1–15 are shown in Supplementary Fig. S6). The *hae-3 hsl2-4* pair of receptors does not abscise at all when present with homozygous mutant or wild-type *EBS3* or *EBS4*. On the other hand, the *hae-5 hsl2-9* pair of receptors does abscise when present with homozygous mutant *ebs3* or *ebs4*, but only partially abscise when present with homozygous wild-type *EBS3* or *EBS4* (Fig. 4B, D, and C, E, respectively). These results suggest that the hae-3 receptor is not responsible for the suppressor phenotype, but the hsl2-9 receptor is probably biochemically functional and is being trafficked to the plasma membrane in the *hae-3 hsl2-9 ebs3 er gl* and the *hae-5 hsl2-9 ebs3* mutants (as well as the *hae-3 hsl2-9 ebs4 er gl* and the *hae-5 hsl2-9 ebs4* mutants).

### HAE and HSL2 are glycoproteins

To test whether HAE and HSL2 are indeed glycoproteins with *N*-glycans, an Endo H assay was used. Endo H is an endoglycosidase capable of cleaving high-mannose-type (H-type) *N*-glycans present on glycoproteins localized in the ER. Endo H cannot, however, cleave Golgi-processed complex-type (C-type) *N*-glycans. Both wild-type HAE and HSL2 receptors were tagged with a C-terminal YFP tag and transformed separately into the *hae-3 hsl2-3* mutant. Tagged wild-type receptors were then treated with Endo H, as well as tagged versions of the mutant receptors hae-3 and hsl2-9 (Fig. 4F, G). Treatment of HAE–YFP with Endo H resulted in the appearance of two distinct bands of slightly different molecular weights (Fig. 4F). This could possibly indicate both an ER-localized receptor (lower molecular weight band) and also a receptor that has been processed by the Golgi (higher molecular weight band). The higher molecular weight band only has a small mobility shift compared with untreated HAE–YFP probably due to the presence of a few incompletely processed *N*-glycans, which has been reported with wild-type BRI1 (Hong *et al.*, 2009). Endo H treatment of HSL–YFP is somewhat less clear, but results in a majority of the protein having a small mobility shift and shows significantly less protein at a lower molecular weight that probably corresponds to ER-localized receptor (Fig. 4G). Both hae-3 and hsl2-9 receptors were tagged with C-terminal YFP and transformed into the *hae-3 hsl2-9 ebs3 er gl* suppressor mutant. Both of these mutant receptors show nearly complete Endo H sensitivity and a greater mobility shift in immunoblot assays, meaning that these mutant receptors are almost completely localized to the ER. Interestingly, a small amount of hsl2-9–YFP was detected at a slightly higher molecular weight than the majority of the Endo H-sensitive ER-localized protein (Fig. 4G). This is likely to indicate that a small amount of receptor is able to escape the ER and be processed by the Golgi, which has also been shown to be the case with bri1-9 in both *ebs3* and *ebs4* suppressor mutants (Hong *et al.*, 2009, 2012). Predictive software was also used to count the number of Asn-Xaa-Ser/Thr sequons present and estimate the number of asparagines likely to be glycosylated on both HAE and HSL2 (R. Gupta *et al.*, unpublished results). Six of the nine sequons in the extracellular domain of HAE were predicted to be glycosylated, and 10 of 14 sequons were predicted to be glycosylated in HSL2 (Supplementary Fig. S7).

### Measuring abundance of hsl2-9 receptor by fluorescent microscopy

**Fig. 5.**
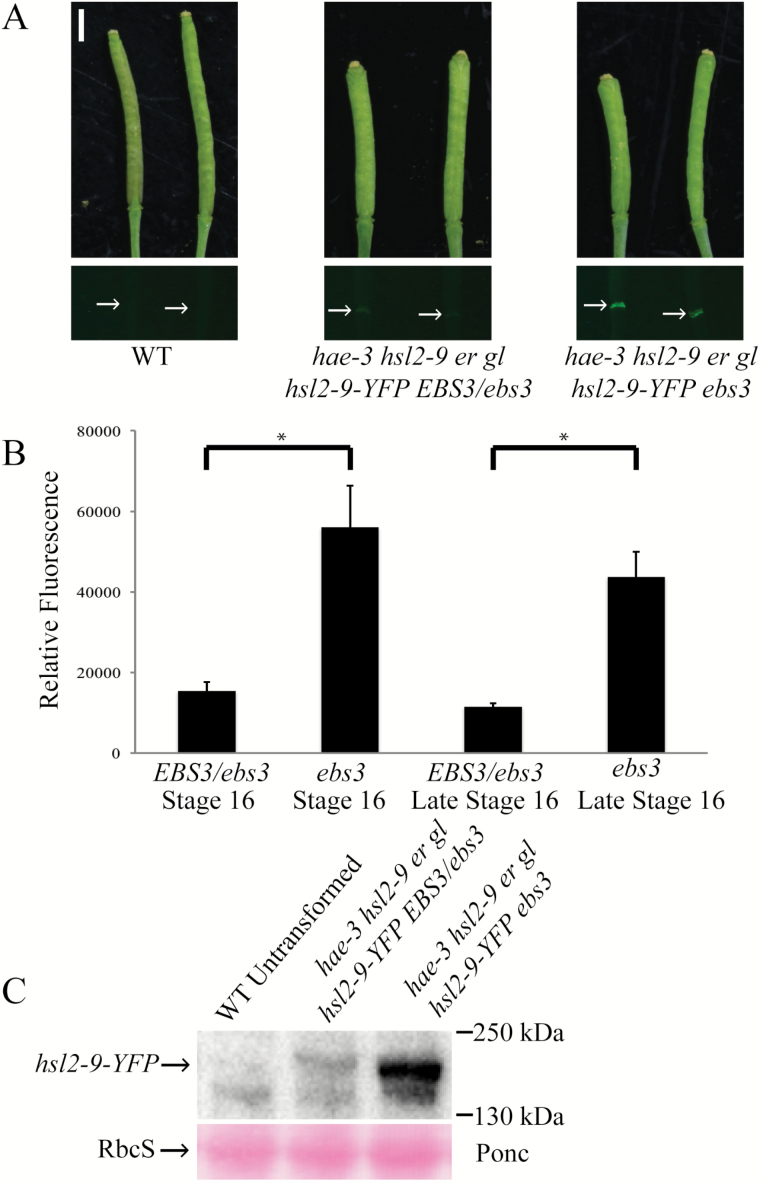
Levels of the hsl2-9–YFP receptor are higher in the *ebs3* mutant. (A) Photographs (white light and YFP fluorescence) of stage 16 and late stage 16 siliques of the wild type, *hae-3 hsl2-9 er gl hsl2-9-YFP EBS3/ebs3*, and *hae-3 hsl2-9 er gl hsl2-9-YFP ebs3.* Floral organs were forcibly removed for all siliques. (B) Quantification of the YFP signal present in abscission zones. Significant differences between EBS3/*ebs3* and *ebs3* for both stage 16 and late stage 16 siliques determined by Student’s *t*-test (*P*-value <0.05, SD error bars shown). (C) Western blot with anti-GFP antibody showing higher detection of hsl2-9–YFP in the *ebs3* mutant than in the *EBS3*/*ebs3* mutant. Bands detected are close to the expected size of hsl2-9–YFP (estimated at 167kDa). Ponceau stain (bottom) for loading control. (This figure is available in colour at *JXB* online.)

To test further the hypothesis that the hsl2-9 mutant receptor is able to escape degradation in the suppressor mutant, but not in the *hae-3 hsl2-9 er gl* parent, we sought to measure the abundance of hsl2-9 protein in both mutant backgrounds by fluorescent microscopy. We added a C-terminal YFP tag to the hsl2-9 mutant receptor and drove expression by the native HSL2 promoter. This was then transformed into *hae-3 hsl2-9 ebs3 er gl*. This experiment was only done in the *ebs3* mutant partly due to time constraints, but primarily because it has already been demonstrated that both *ebs3* and *ebs4* mutants accumulate more *bri1-9,* and both of these suppressors were expected to behave similarly in regards to disrupting ERAD and accumulating protein (Hong *et al.*, 2012). Expressing T_1_ lines were then crossed to the *hae-3 hsl2-9 er gl* parent and to *hae-3 hsl2-9 ebs3 er gl*. The F_1_ progeny of these crosses, being either *EBS3*/*ebs3* (containing one functional copy of *EBS3*) or *ebs3* (homozygous for the suppressor mutation), were checked for YFP expression. Only F_1_ progeny that came from a single expressing T_1_ plant were compared to control for any differences in expression that could occur between T_1_s. Photographs of YFP expression in the abscission zones of stage 16 and late stage 16 siliques show a clear difference in signal between the two crosses (Fig. 5A), with the *ebs3* progeny having significantly higher YFP signal than *EBS3*/*ebs3*, which is consistent with our hypothesis that hsl2-9 receptor can escape degradation in the *ebs3* suppressor mutant, but not in the *hae-3 hsl2-9 er gl* parent (*EBS3*). Both stage 16 and late stage 16 siliques were measured in order to give two time points during the process of abscission. Photographs of YFP expression were then quantified using ImageJ software, which assigns values to pixels depending on the color displayed (i.e. amount of fluorescent signal), and averages the values of pixels within a defined region. For these images, this region was carefully traced around abscission zones of individual siliques, allowing for signal to be measured and then normalized to the wild type (Fig. 5B). Samples were then analyzed by western blot to confirm that full-length hsl2-9–YFP receptor was present. Probing with anti-GFP antibody revealed bands close to the expected size of hsl2-9–YFP (estimated to be 167kDa), and showed a similar pattern of protein levels, with *ebs3* having a higher level of hsl2-9–YFP than *EBS3/ebs3* (Fig. 5C). This experiment was repeated using YFP-tagged hae-3 receptor. Fluorescent microscopy revealed an identical pattern of signal, with the *EBS3/ebs3* mutant showing significantly less YFP signal than *ebs3* (Supplementary Fig. S8), suggesting that hae-3 is also likely to be subject to ERAD. Because the hae-3–YFP mutant receptor is non-functional in both the mutant *ebs3* background and the *EBS3/ebs3* background, this suggests that the *hae-3* mutation causes a negative impact on receptor function in addition to ERAD (Fig. 4).

### Hypothetical model of ERQC and ERAD

**Fig. 6.**
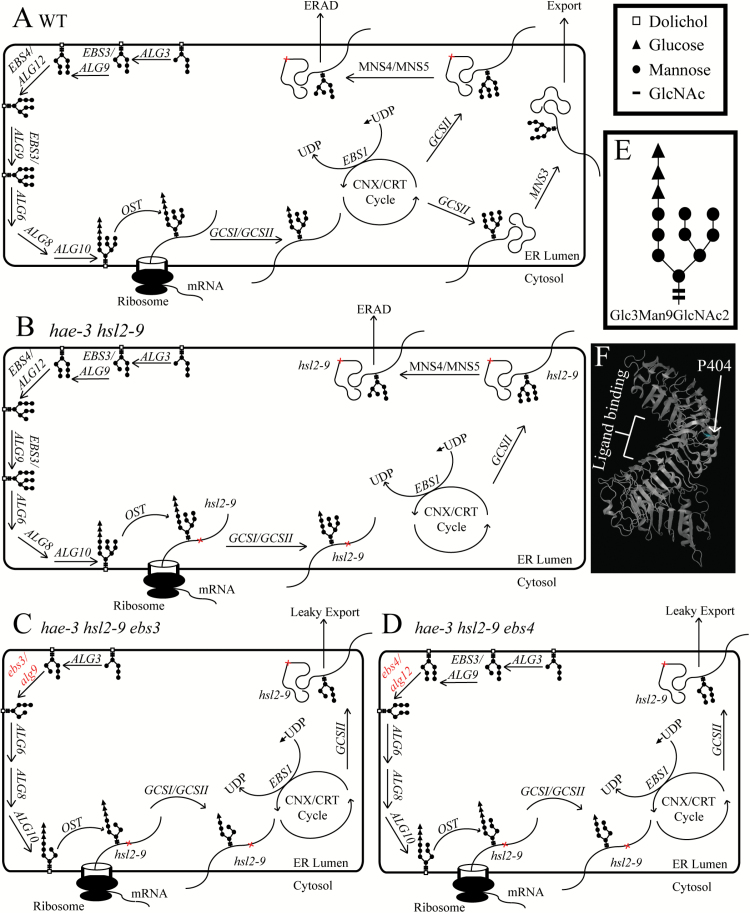
Hypothetical model of *N*-glycan assembly and protein folding. (A) Wild-type cells sequentially add mannose and glucose molecules to assemble the *N*-glycan Glc_3_Man_9_GlcNAc_2_ in the ER. Glycans are then transferred onto nascent polypeptides before entering the CNX/CRT cycle of protein folding. Correctly folded proteins are eventually exported, while terminally misfolded proteins are sent for degradation (both of these paths are dependent upon trimming *N*-glycans to expose specific mannose residues). (B) The *hae-3 hsl2-9* mutant correctly assembles Glc_3_Man_9_GlcNAc_2_, and polypeptides enter the protein folding cycle, but structural imperfections in hsl2-9 prevent correct folding (note that hae-3 is also likely to be misfolded as well). These proteins will be sent for degradation. (C, D) The *hae-3 hsl2-9 ebs3* (C) and *hae-3 hsl2-9 ebs4* (D) mutants do not assemble complete *N*-glycans, preventing α1,6-linked mannose residues from being exposed to signal receptors for degradation. This allows mutant hsl2-9 receptor to leak out of the ER and be trafficked to the plasma membrane to signal to regain abscission. Note that the *ebs3* mutation prevents downstream action of *EBS4*, and assembly of the glycan is halted (C). Similarly, the *ebs4* mutation prevents *EBS3* from adding a second mannose residue, also halting glycan assembly (D). (E) Structure of Glc_3_Man_9_GlcNAc_2_. (F) Theoretical structure of the HSL2 receptor showing the P404 residue and putative ligand-binding region. (This figure is available in colour at *JXB* online.)

Figure 6A illustrates normal ERQC and ERAD systems. Note that this model is partially based on data from studies in yeast, as ERQC and ERAD have not been studied in plants as extensively. In wild-type cells, the glycan precursor Glc_3_Man_9_GlcNAc_2_ (where Glc is glucose, Man is mannose, and GlcNAc is *N*-acetylglucosamine, shown in Fig. 6E) is assembled in the ER and transferred onto asparagine residues of nascent polypeptides contained in the Asn-X-Ser/Thr motif (where X represents any residue except proline). Two glucose molecules are then trimmed from Glc_3_Man_9_GlcNAc_2_ by α-glucosidase I (GCSI) and α-glucosidase II (GCSII), and polypeptides now carry Glc_1_Man_9_GlcNAc_2_, which is recognized by calnexin (CNX) and calreticulin (CRT) to enter the protein folding cycle. Proteins are released from this cycle when GCSII cleaves the final glucose, leading to either export or re-entry into the protein folding cycle if correct folding was not achieved. In the case of re-entry and continued folding, *N*-glycans are re-glucosylated by UDP-Glc:glycoprotein glucosyltransferase (UGGT, or EBS1 in Arabidopsis) to allow CNX/CRT to re-bind the protein and attempt to fold correctly. This cycle of attempted protein folding will continue for a time; however, if proper folding is never achieved, proteins will have the remaining glucose and two mannose molecules trimmed from Glc_1_Man_9_GlcNAc_2_ by GCSII and MNS4/MNS5, respectively. The resulting *N*-glycans have an exposed α1,6-linked mannose present on the C-branch, which is the signal for degradation (Clerc *et al.*, 2009). This is the case with mutant receptors that are structurally compromised (i.e. hae-3, hsl2-9, or bri1-9), and they will only be sent for degradation, as they never achieve proper folding (Fig. 6B). Note that in Fig. 6B–D, only hsl2-9 is shown to be misfolded. The hae-3 receptor is also probably misfolded and subject to ERAD (Supplementary Fig. S8), but is not shown because only the hsl2-9 receptor is involved in regaining abscission (Fig. 4).

The *ebs3* and *ebs4* suppressor mutants (Fig. 6C, D) differ in these processes because the glycans they assemble are incomplete (Hong *et al.*, 2012). The *ebs3* mutation halts the assembly of glycans and prevents downstream action of *EBS4*, resulting in incomplete glycans. Likewise, the *ebs4* mutation prevents the downstream action of *EBS3*, and glycan assembly cannot continue. In both of these mutants, glycans are still transferred onto polypeptides, which then enter the CNX/CRT cycle to attempt folding. Mutant receptors will never achieve proper folding due to structural imperfections, but here they cannot be sent for degradation because their glycans cannot be properly trimmed to expose the α1,6-linked mannose. This will hypothetically result in retention and accumulation of these proteins in the ERQC/protein folding machinery until this system is oversaturated and some protein is likely to be able to escape the ER and be sent to its proper destination. Here, these proteins can bind ligand and regain signaling if biochemically functional, as is probably the case with hsl2-9. A theoretical structure of HSL2 (Fig. 6F) based on the known structure of BRI1 maps the P404 residue to the opposite side of the extracellular domain to the putative ligand-binding region, and supports the hypothesis that hsl2-9 can still function because it is unlikely that the hsl2-9 lesion (P404L) would interfere with ligand binding.

## Discussion

In this study, we identify two mutant lines that show suppression of the abscission defect present in the parental *hae-3 hsl2-9 er gl* mutant. Both of these lines were isolated from an EMS screen of *hae-3 hsl2-9 er gl*. The genomes of both of these suppressors were sequenced to reveal the most likely candidate genes, *EBS3* and *EBS4*, causing the suppressor phenotype. It was then confirmed by complementation that the suppressor phenotypes of these mutants are caused by mutations in the genes *EBS3* and *EBS4*. *EBS3* encodes the Arabidopsis ortholog of the yeast gene *ASPARAGINE-LINKED GLYCOSYLATION 9* (*ALG9*) (Hong *et al.*, 2012), and catalyzes the addition of two α1,2 mannose (man) residues during the assembly of *N*-glycans in the lumen of the ER. *EBS4*, also involved in the ER luminal assembly of *N*-glycans, encodes the ortholog of the yeast gene *ASPARAGINE-LINKED GLYCOSYLATION 12* (*ALG12*) (Hong *et al.*, 2009), and catalyzes the addition of an α1,6 man residue. These genes are only two components of the highly specific assembly of the three branched *N*-glycans (Glc_3_Man_9_GlcNAc_2_) that are heavily utilized in ERQC and ERAD. Failure at any one of the steps in assembling Glc_3_Man_9_GlcNAc_2_ can result in incomplete or incorrectly constructed *N*-glycans that are incapable of being recognized by ERQC and ERAD machinery (Liu and Li, 2014; Lannoo and Van Damme, 2015).

It has been previously shown that making loss-of-function mutations in many of the mannosyltransferases involved in assembling Glc_3_Man_9_GlcNAc_2_ can suppress mutant phenotypes, most notably the semi-dwarf phenotype of weak *bri1* alleles (Jin *et al.*, 2007, 2009; Hong *et al.*, 2009, 2012). Here, we demonstrate that *ebs3* and *ebs4* mutants are able to suppress the partial abscission defect of *hae-3 hsl2-9 er gl* in a similar manner, which connects ERQC, ERAD, and the strict regulation of protein folding to the process of abscission. As the receptors HAE and HSL2 are being produced, these methods of regulation are in place to prevent toxic or non-functional proteins from accumulating in the cell. We also demonstrate that these methods are often overly stringent in sending incompletely or incorrectly folded proteins for degradation, as many mutant receptors are biochemically functional despite minor imperfections or lesions affecting their structure. This is clearly the case with hsl2-9, which can function solely to regain abscission (Fig. 4B, C) when not impeded by degradation. We also further characterized the *ebs3* mutant by looking at protein levels of fluorescently tagged hsl2-9 receptor. Fluorescent signal was observed to have a significant increase in the *ebs3* mutant compared with *EBS3/ebs3* (Fig. 5A, B), suggesting that hsl2-9 is able to escape degradation and accumulate in the suppressor, but that much of the receptor is degraded when at least one functional copy of *EBS3* is present and ERAD is still functional.

## Supplementary data

Supplementary data are available at *JXB* online.

Figure S1. Allele diagrams, range of abscission phenotypes, and average number of petals and sepals retained for *er gl*, *hae-3 hsl2-3 er gl*, *hae-3 hsl2-9 er gl*, *hae-3 hsl2-9 er gl* Line 1.6, and *hae-3 hsl2-9 er gl* Line 3.3.

Figure S2. Pictures of the brush device used for quantification of abscission phenotypes, including dimensions and method of use.

Figure S3. Graphs showing the proportion of reads containing an SNP for each variant across all five chromosomes and all pools of DNA sequenced.

Figure S4. PCR flanking introns with a suppressor mutation shows incorrect splicing.

Figure S5. Average number of petals and sepals retained for complemented lines containing the *gEBS3* or *gEBS4* transgene.

Figure S6. Average number of petals and sepals retained for *ebs3* and *ebs4* mutants crossed to the double null mutant *hae-5 hsl2-4.*

Figure S7. Predicted number and location of *N*-glycans in HAE and HSL2 extracellular domains.

Figure S8. Levels of hae-3–YFP receptor are higher in the *ebs3* suppressor mutant.

File S1. General work flow for genome sequencing data alignment and analysis, resulting in a list of variants. Includes commands entered with descriptions.

File S2. List of candidate genes from linked region of chromosome 1 for line 1.6 and line 3.3.

File S3. List of primer sequences and descriptions.

Supplementary Data
