# Supplementary material for: Disrupting ER-associated protein degradation suppresses the abscission defect of a weak hae hsl2 mutant in Arabidopsis
Source: J Exp Bot. 2016 Aug 26;67(18):5473–84. doi: 10.1093/jxb/erw313 (PMC5049395; doi:10.1093/jxb/erw313)
Supplement: Supplementary Data [file supp_67_18_5473__index.html]

Disrupting ER-associated protein degradation suppresses the abscission defect of a weak hae hsl2 mutant in Arabidopsis — Disrupting ER-associated protein degradation suppresses the abscission defect of a weak hae hsl2 mutant in Arabidopsis — Supplementary Data 

# Disrupting ER-associated protein degradation suppresses the abscission defect of a weak *hae hsl2* mutant in Arabidopsis

## Supplementary Data

Data files

- Supplementary\_file\_1.docx - Supplementary Data
- Supplementary\_file\_2.xlsx - Supplementary Data
- Supplementary\_file\_3.xlsx - Supplementary Data
- Supplementary\_figures\_S1\_S8.pdf - Supplementary Data
