# Supplementary material for: Disrupting ER-associated protein degradation suppresses the abscission defect of a weak hae hsl2 mutant in Arabidopsis
Source: J Exp Bot. 2016 Aug 26;67(18):5473–84. doi: 10.1093/jxb/erw313 (PMC5049395; doi:10.1093/jxb/erw313)
Supplement: Supplementary Data [file supp_erw313_Supplementary_file_1.docx]

Work Flow

**-Downloaded Arabidopsis genome TAIR10_chr_all.fas from** <ftp://ftp.arabidopsis.org/home/tair/Genes/TAIR10_genome_release/TAIR10_chromosome_files/>

**-Built database from genome file that bowtie2 can use:**

mu-080146:JB_h3h9_suppressor_mapping iwtwb8$ ../Illumina_data/bowtie2/bowtie2-build TAIR10_chr_all.fas TAIR10

-bowtie2-build command builds database

-Uses “TAIR10_chr_all.fas”

-Output files are all titled “TAIR10”

**-Aligned both 1.6 suppressor files to TAIR10 database:**

-The –p 4 uses all 4 processors of the computer (aligns in about 58 minutes as opposed to about 3 hours and 19 minutes when using one core)

-x TAIR10 gives the database to be used

-1 <Name of first read file>

-2 <Name of second read file>

-S names output file

mu-080146:JB_h3h9_suppressor_mapping iwtwb8$ ../Illumina_data/bowtie2/bowtie2 -p 4 -x TAIR10 -1 ../Illumina_data/fastq_files/1_6-supp_CTTGTA_L002_R1_001.fastq -2 ../Illumina_data/fastq_files/1_6-supp_CTTGTA_L002_R2_001.fastq -S JB_1_6-supp_p4_TAIR10.sam

59413479 reads; of these:

59413479 (100.00%) were paired; of these:

10600491 (17.84%) aligned concordantly 0 times

37330705 (62.83%) aligned concordantly exactly 1 time

11482283 (19.33%) aligned concordantly >1 times

----

10600491 pairs aligned concordantly 0 times; of these:

6385019 (60.23%) aligned discordantly 1 time

----

4215472 pairs aligned 0 times concordantly or discordantly; of these:

8430944 mates make up the pairs; of these:

3107231 (36.86%) aligned 0 times

1700119 (20.17%) aligned exactly 1 time

3623594 (42.98%) aligned >1 times

97.39% overall alignment rate

**-Aligned the 1.6 non-suppressor files to TAIR10 database:**

-Uses same commands as previous alignment

mu-080146:JB_h3h9_suppressor_mapping iwtwb8$ ../Illumina_data/bowtie2/bowtie2 -p 4 -x TAIR10 -1 ../Illumina_data/fastq_files/1_6-non_GTGAAA_L002_R1_001.fastq -2 ../Illumina_data/fastq_files/1_6-non_GTGAAA_L002_R2_001.fastq -S JB_1_6-non_p4_TAIR10.sam

30227306 reads; of these:

30227306 (100.00%) were paired; of these:

5352117 (17.71%) aligned concordantly 0 times

19050429 (63.02%) aligned concordantly exactly 1 time

5824760 (19.27%) aligned concordantly >1 times

----

5352117 pairs aligned concordantly 0 times; of these:

3140218 (58.67%) aligned discordantly 1 time

----

2211899 pairs aligned 0 times concordantly or discordantly; of these:

4423798 mates make up the pairs; of these:

1795683 (40.59%) aligned 0 times

867059 (19.60%) aligned exactly 1 time

1761056 (39.81%) aligned >1 times

97.03% overall alignment rate

**-Aligned the 3.3 suppressor files to TAIR10 database:**

-Uses same commands as previous alignment

mu-080146:JB_h3h9_suppressor_mapping iwtwb8$ ../Illumina_data/bowtie2/bowtie2 -p 4 -x TAIR10 -1 ../Illumina_data/fastq_files/3_3-supp_GCCAAT_L002_R1_001.fastq -2 ../Illumina_data/fastq_files/3_3-supp_GCCAAT_L002_R2_001.fastq -S JB_3-3_supp_p4_TAIR10.sam

52094616 reads; of these:

52094616 (100.00%) were paired; of these:

9160960 (17.59%) aligned concordantly 0 times

32299007 (62.00%) aligned concordantly exactly 1 time

10634649 (20.41%) aligned concordantly >1 times

----

9160960 pairs aligned concordantly 0 times; of these:

5427467 (59.25%) aligned discordantly 1 time

----

3733493 pairs aligned 0 times concordantly or discordantly; of these:

7466986 mates make up the pairs; of these:

3021393 (40.46%) aligned 0 times

1506244 (20.17%) aligned exactly 1 time

2939349 (39.36%) aligned >1 times

97.10% overall alignment rate

**-Converted .sam files to .bam**

mu-080146:JB_h3h9_suppressor_mapping iwtwb8$ ../Illumina_data/samtools-1.1/samtools view -bS JB_1_6-supp_p4_TAIR10.sam >JB_1_6-supp_p4_TAIR10.bam

-“samtools view” converts .sam files to .bam

-This was repeated for 1.6 non-suppressor and 3.3 suppressor alignment files

-**Converted .bam files to sorted bam files (.sorted)**

mu-080146:JB_h3h9_suppressor_mapping iwtwb8$ ../Illumina_data/samtools-1.1/samtools sort JB_1_6-supp_p4_TAIR10.bam JB_1_6-supp_p4_TAIR10.sorted

-“samtools sort” converts .bam files to sorted bam files (.sorted)

-This was repeated for 1.6 non-suppressor and 3.3 suppressor

**-Used mpileup command**

mu-080146:JB_h3h9_suppressor_mapping iwtwb8$ ../Illumina_data/samtools-1.1/samtools mpileup -g -f TAIR10_chr_all.fas JB_1_6-supp_p4_TAIR10.sorted.bam > JB_1_6-supp-pileup.bcf

[mpileup] 1 samples in 1 input files

<mpileup> Set max per-file depth to 8000

-“mpileup” produces a table displaying coverage at each position

-This was repeated for 1.6 non-suppressor and 3.3 suppressor

**-Indexed the .sorted.bam files**

mu-080146:JB_h3h9_suppressor_mapping iwtwb8$ ../Illumina_data/samtools-1.1/samtools index JB_1_6-supp_p4_TAIR10.sorted.bam

-“samtools index” creates the indexed .sorted.bam file needed by IGV

- This was repeated for 1.6 non-suppressor and 3.3 suppressor

-Once indexed bam files are created, IGV can be used to visualize data

**-Call variants**

mu-080146:JB_h3h9_suppressor_mapping iwtwb8$ ../Illumina_data/samtools-1.1/bcftools/bcftools call -mv JB_1_6-supp-pileup.bcf >JB_1_6-supp.vcf

-“bcftools call” calls all variants, this replaced the “bcftools view” command that was in previous versions

-“-m” uses multiallelic caller, as opposed to “-c” (consensus caller)

-Input is the .bcf file created from the “samtools mpileup” command

-This was repeated for 1.6 non-suppressor and 3.3 suppressor

**-Select lines with G->A or C->T**

mu-080146:JB_h3h9_suppressor_mapping iwtwb8$ grep ' G A ' JB_3_3-supp.vcf >GA_temp

-This searches the “JB_3_3-supp.vcf” file for lines with only G->A and prints the whole line to a temporary file

mu-080146:JB_h3h9_suppressor_mapping iwtwb8$ grep ' C T ' JB_3_3-supp.vcf >CT_temp

-This searches the “JB_3_3-supp.vcf” file for lines with only C->T and prints the whole line to a temporary file

mu-080146:JB_h3h9_suppressor_mapping iwtwb8$ cat GA_temp CT_temp >JB_3_3-supp-GA_CT_vcf

-This combines both temporary files previously created to make one file containing every line with either G->A or C->T. These files were then imported into excel.

**-Imported variant files previously generated into excel**

-Imported data from text files, delineated by Tab

-Sorted all by Chromosome number and by position

-DP4 numbers are number of high quality ref-forward, ref-reverse, alt-forward, and alt-reverse

-Used command to cut out section to the right of the “DP4=”, put into new cell

-Cut everything to the left of the “;” which should be only the four DP4 numbers

-Used logical statement to determine which lines had DP4 sum greater than 15 and less than 100. Lines with values outside this range were returned as empty cells. Then calculated (alt-forward + alt-reverse)/(ref-forward + ref-reverse) to be plotted for each chromosome.
