# Supplementary material for: Disrupting ER-associated protein degradation suppresses the abscission defect of a weak hae hsl2 mutant in Arabidopsis
Source: J Exp Bot. 2016 Aug 26;67(18):5473–84. doi: 10.1093/jxb/erw313 (PMC5049395; doi:10.1093/jxb/erw313)
Supplement: Supplementary Data [file supp_erw313_Supplementary_figures_S1_S8.pdf]

Supplementary Figures:

Supplementary Figure 1:

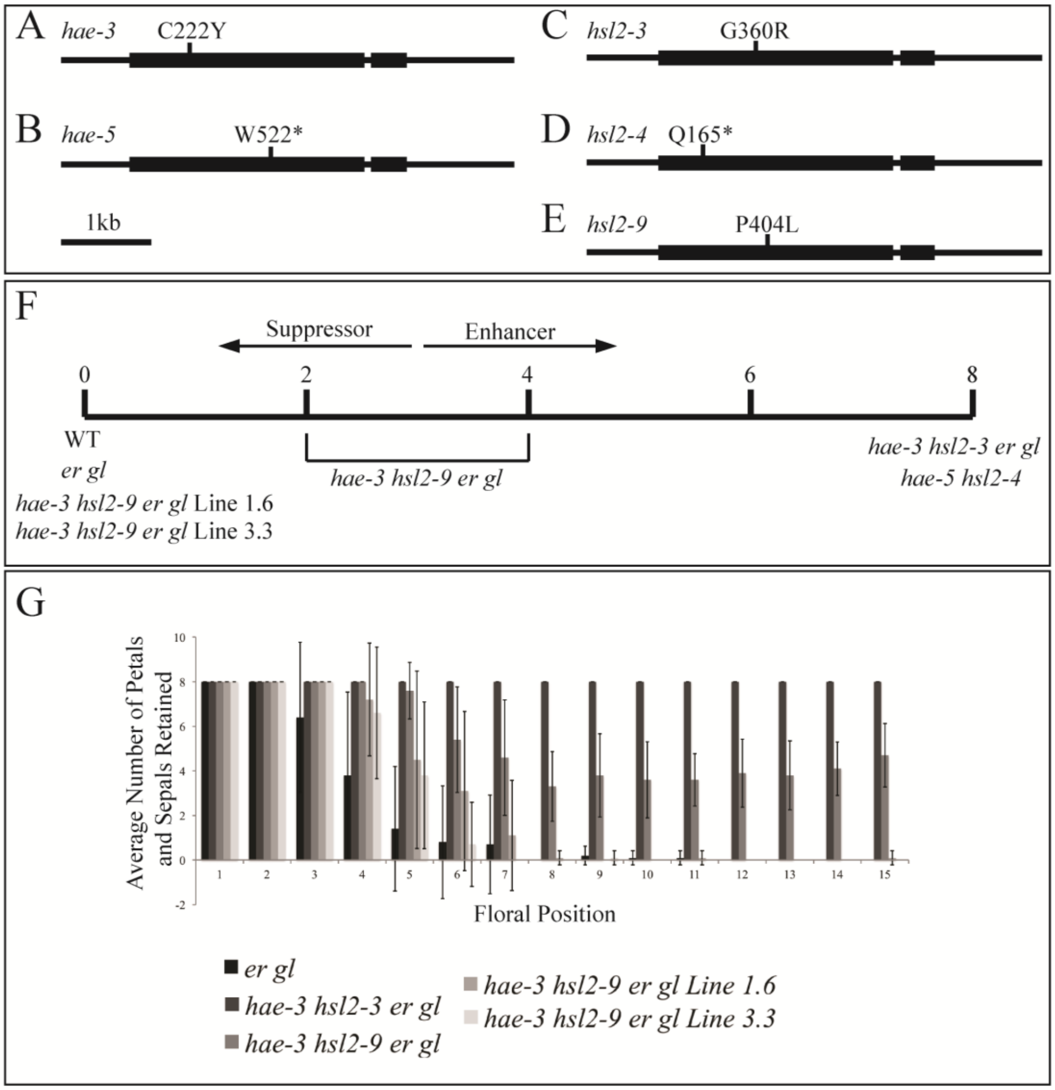

Supplementary Figure 1: Allele diagrams, range of abscission phenotypes, and average number of petals and sepals retained

A-E) Lesions present in relevant alleles of *hae* and *hsl2*. A) *hae-3*, B) *hae-5*, C) *hsl2-3*, D) *hsl2-4*, and E) *hsl2-9*. F) Range of abscission phenotypes of various mutants. G) Average number of petals and sepals retained for positions 1-15 for *er gl*, *hae-3 hsl2-3 er gl*, *hae-3 hsl2-9 er gl*, *hae-3 hsl2-9 er gl* Line 1.6, *hae-3 hsl2-9 er gl* Line 3.3 (n=10, St. Dev. error bars shown).

Supplementary Figure 2:

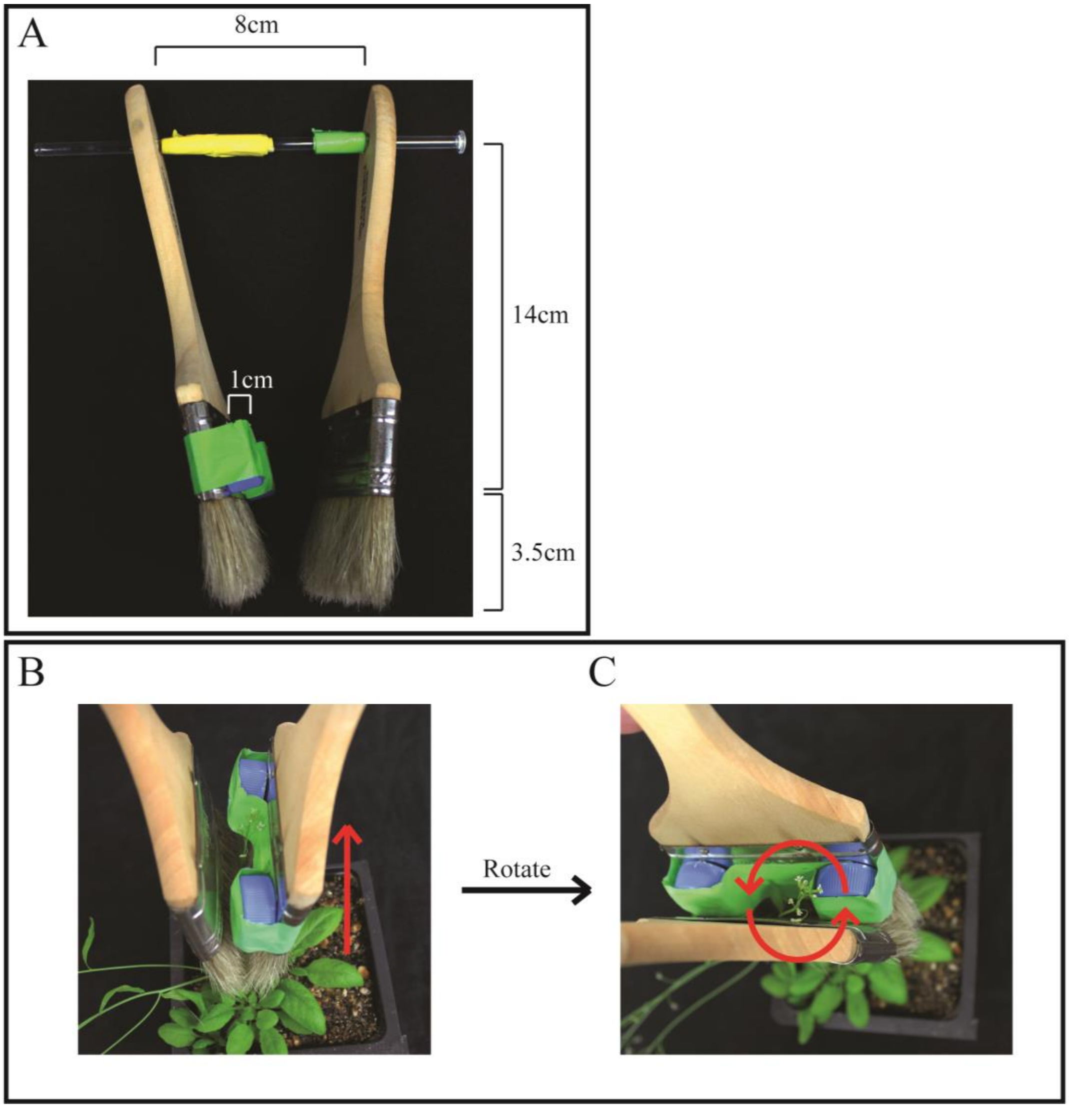

Supplementary Figure 2: Pictures of brush device used for quantification of abscission phenotypes, including dimensions and method of use

Brush device used for quantification of abscission phenotypes. A) dimensions of brush device. B) First treatment with brush device. Inflorescence is placed between brushes and device is pulled upward to treat entire inflorescence. C) Second treatment with brush device. Device is rotated ninety degrees around inflorescence (arrows show direction of rotation prior to second treatment) and pulled upward again to repeat treatment.

Supplementary Figure 3:

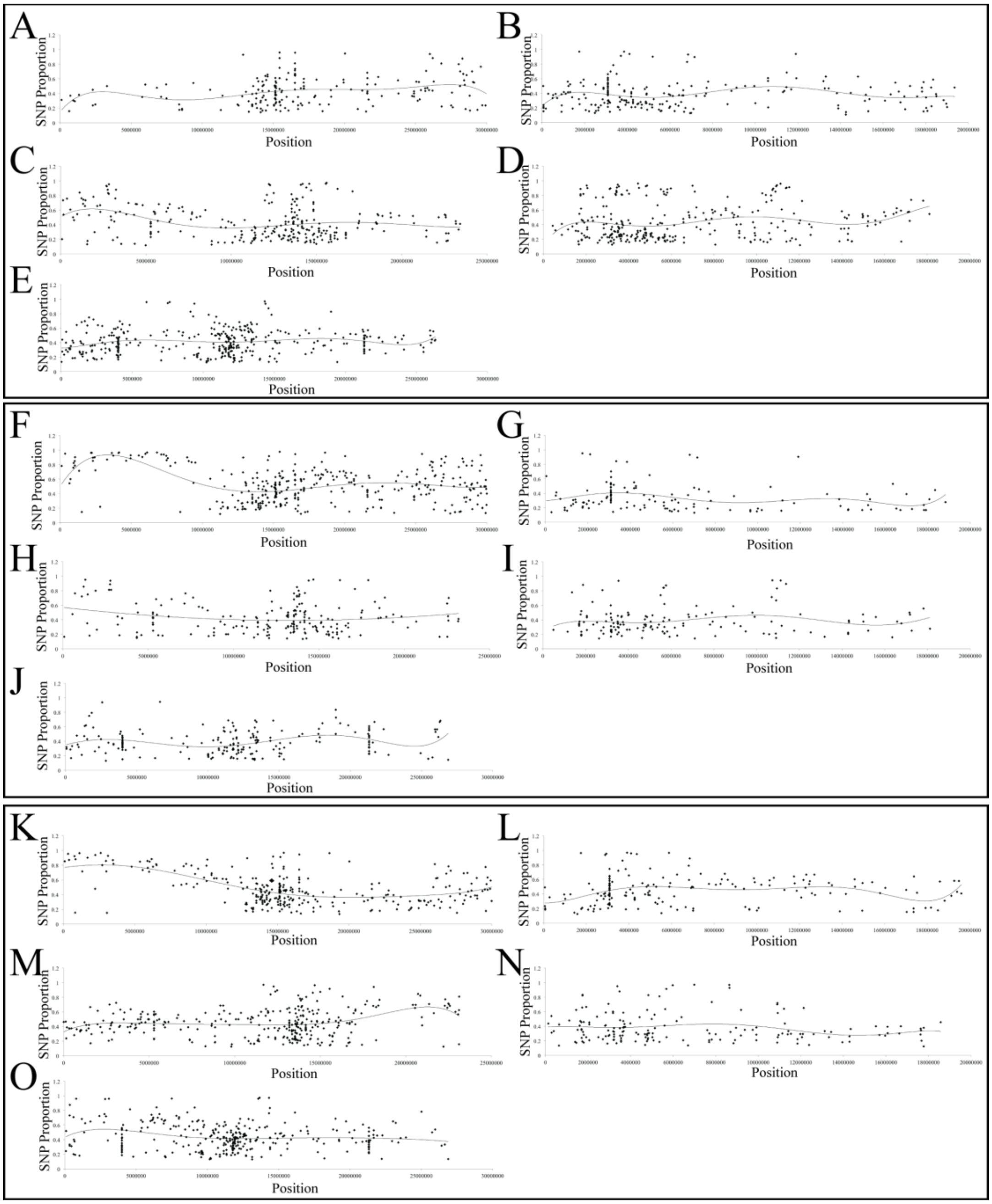

Supplementary Figure 3: Graphs showing proportion of reads containing SNP for each pool

Graphs of SNP Proportion for variants by chromosomal position for A-E) pool of non-suppressed individuals from F2 of line 1.6 backcross, F-J) pool of suppressed individuals from F2 of line 1.6 backcross, and K-O) pool of suppressed individuals from F2 of line 3.3 backcross. Chromosomes one through five shown sequentially for all pools.

Supplementary Figure 4:

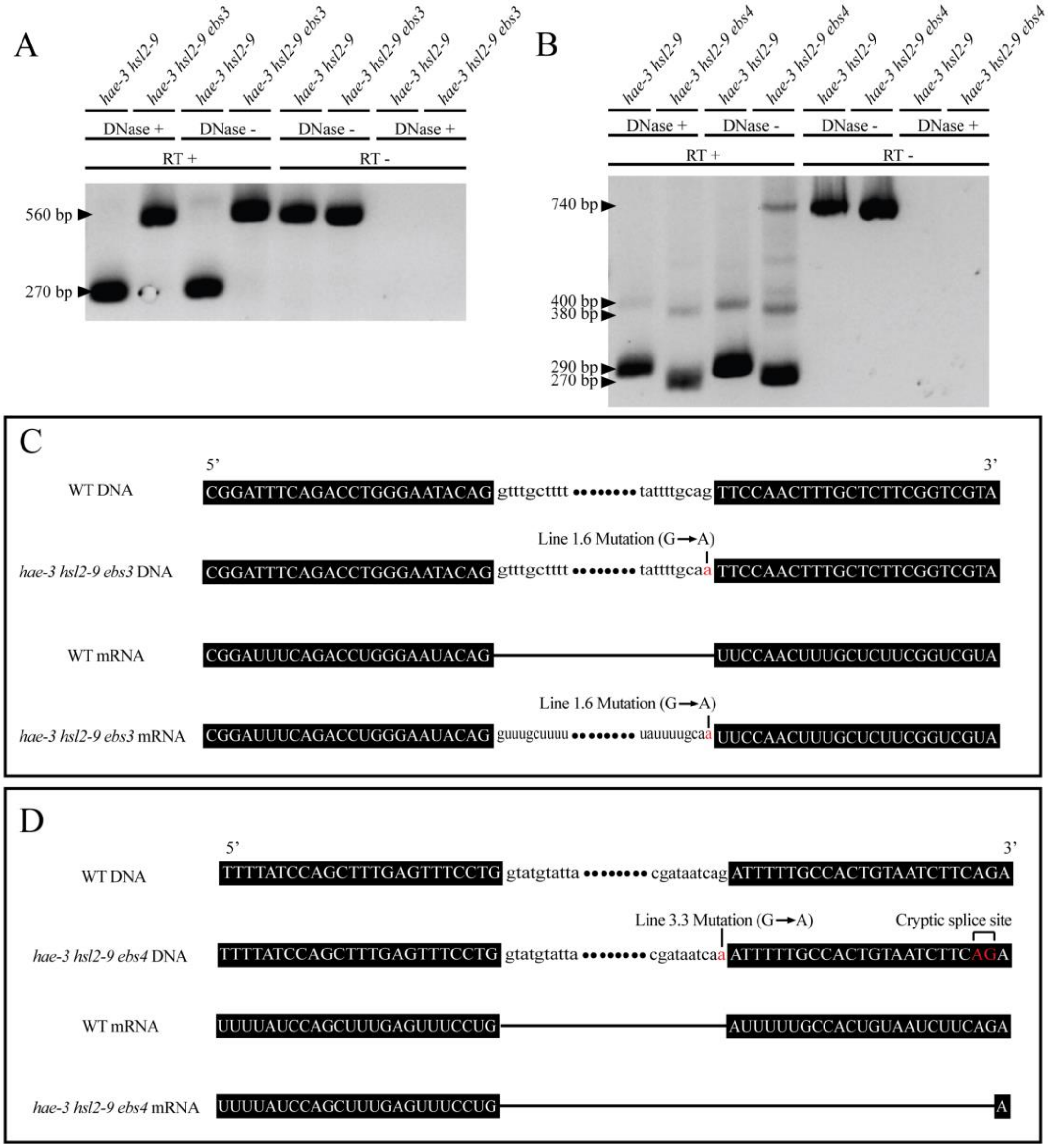

Supplementary Figure 4: PCR flanking introns with suppressor mutation shows improper splicing

A) Gel of PCR flanking intron containing mutation in line 1.6. Product from *ebs3* mutant is approximately 290 base pairs larger than that from *hae-3 hsl2-9* parent, suggesting retention of intron. B) Gel of PCR flanking intron containing mutation in line 3.3. Product from *ebs4* mutant is approximately 20bp shorter than that from *hae-3 hsl2-9* parent. Sequencing revealed activation of a cryptic splice site downstream of mutation. C) Sequence alignments of wild type and *ebs3* DNA and mRNA, showing line 1.6 mutation and intron retained in *ebs3.* D) Sequence alignments of wild type and *ebs4* DNA and mRNA, showing line 3.3 mutation, cryptic splice site, and additional fragment spliced from *ebs4.*

Supplementary Figure 5:

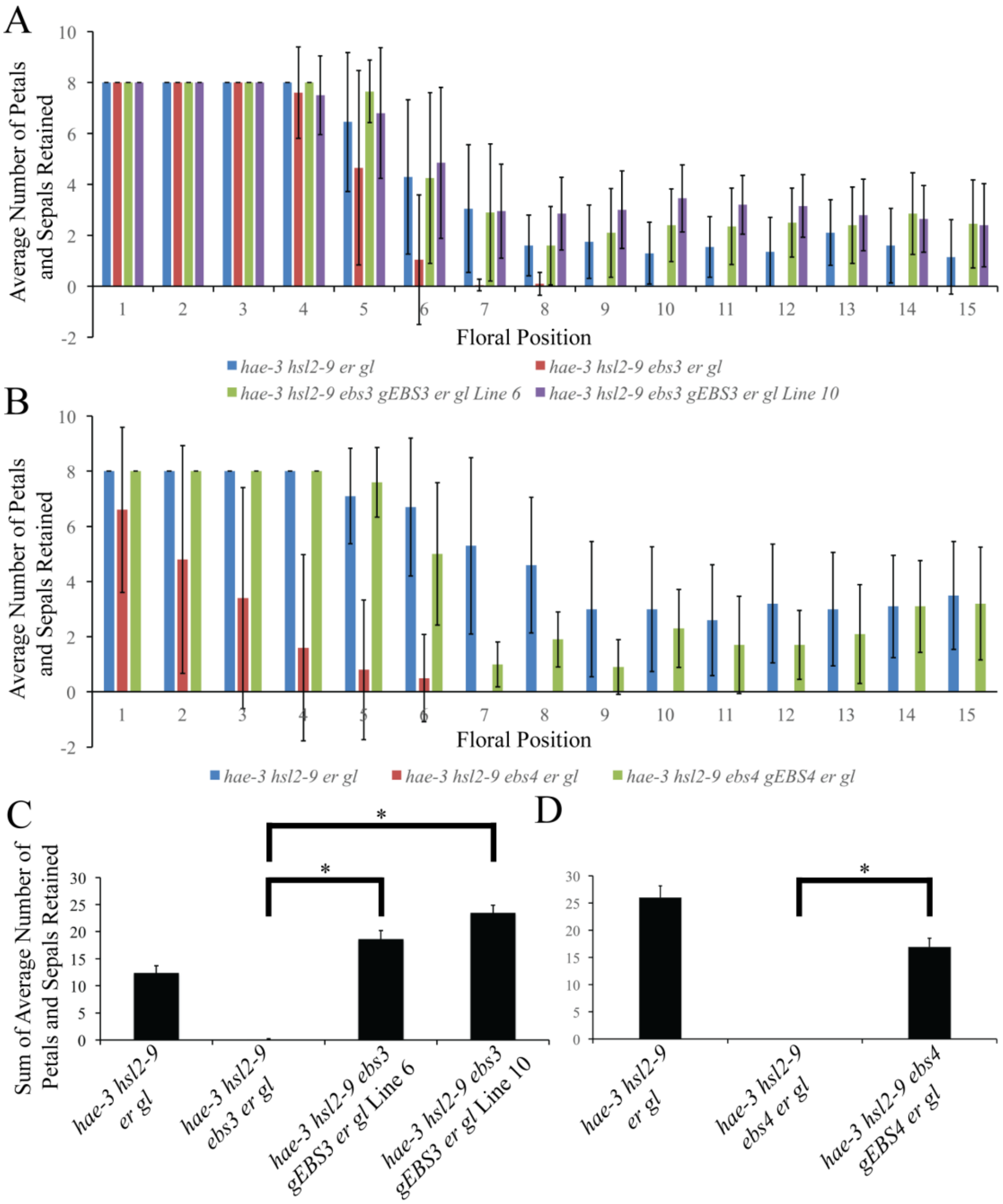

Supplementary Figure 5: Average number of petals and sepals retained for complemented lines containing *gEBS3* or *gEBS4* transgene

Average number of petals and sepals retained for positions 1-15 for A) *hae-3 hsl2-9 er gl*, *hae-3 hsl2-9 ebs3 er gl*, *hae-3 hsl2-9 ebs3 gEBS3 er gl* T2 Lines 6 and 10 (n=20), and B) *hae-3 hsl2-9 er gl*, *hae-3 hsl2-9 ebs4 er gl*, *hae-3 hsl2-9 ebs4 gEBS4 er gl* T1s (n=10). Sum of average number of petals and sepals retained for positions 8-15 for C) *gEBS3* T2 Lines 6 and 10, and D) *gEBS4* T1s (statistical significance determined by Student's t-test, p-value <0.05, St. Dev. error bars shown for all).

Supplementary Figure 6:

A

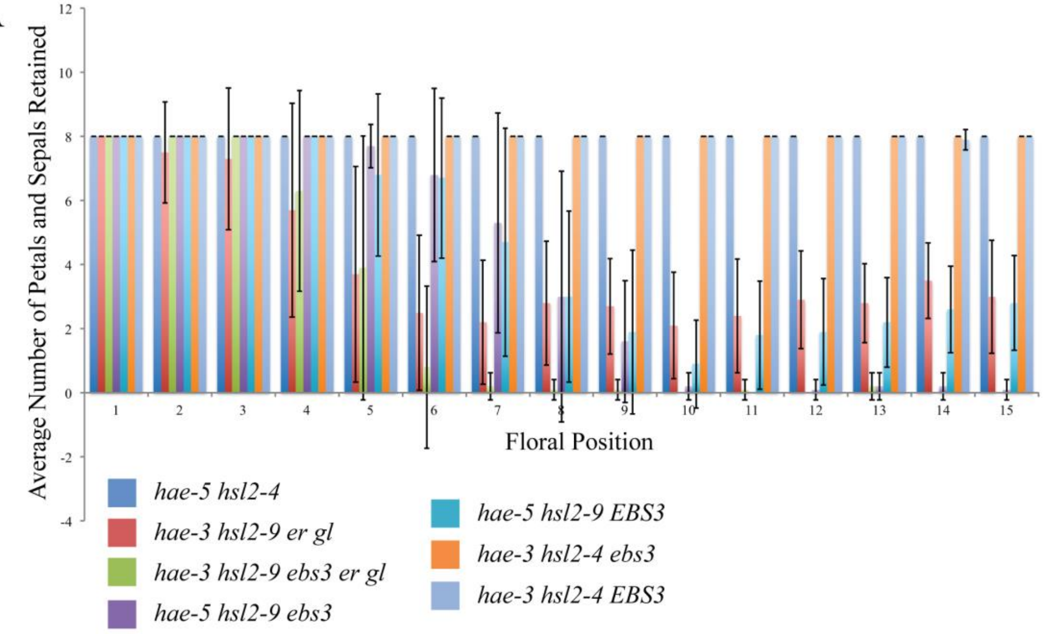

B

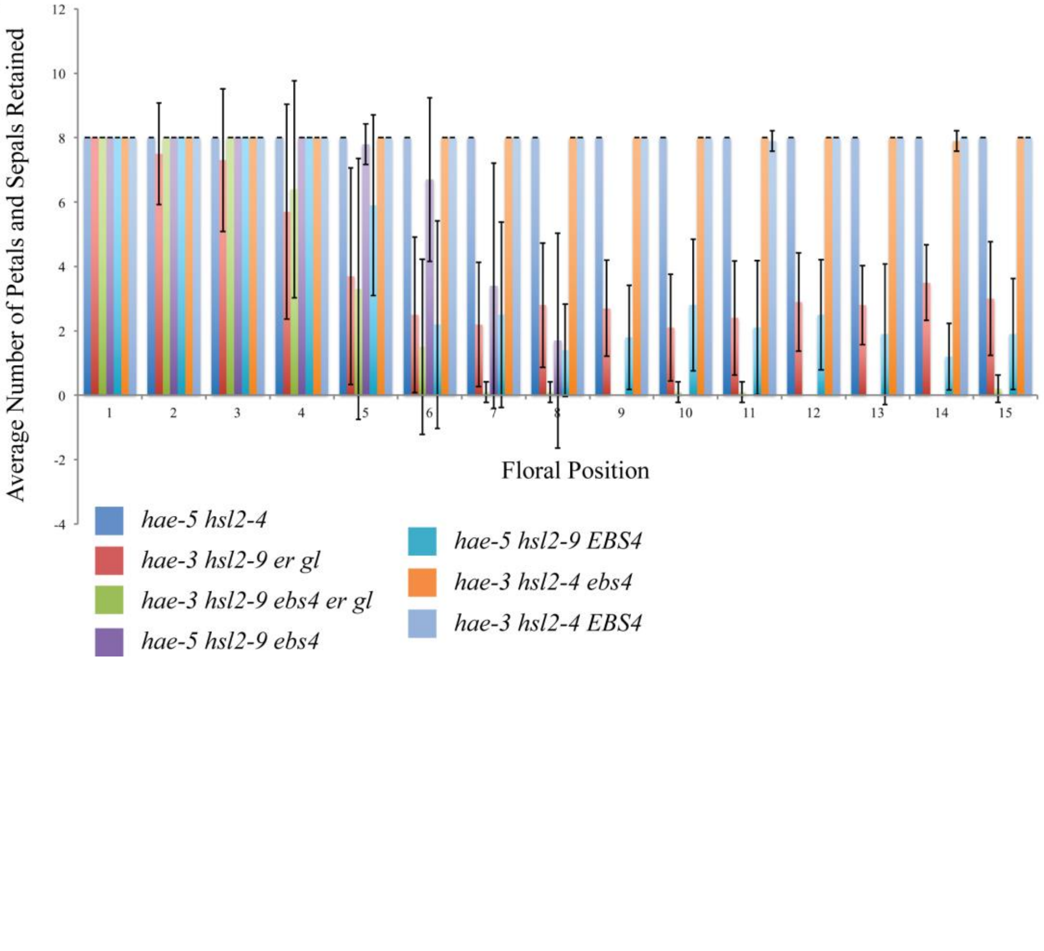

Supplementary Figure 6: Average number of petals and sepals retained for *ebs3* and *ebs4* mutants crossed to *hae-5 hsl2-4*

Average number of petals and sepals retained for positions 1-15 for A) *hae-3 hsl2-9 ebs3* mutant crossed to double null mutant *hae-5 hsl2-4*, and B) *hae-3 hsl2-9 ebs4* mutant crossed to double null mutant *hae-5 hsl2-4* (n=10, St. Dev. error bars shown for all).

Supplementary Figure 7:

A HAE extracellular domain

```
MLYCLILLLCLSSTYLPSLSLNQDATILRQAKLGLSDPAQSLSSWSDNNDVTPCKWLGVSCDATSNVVSVDLSSFMLVGP   80
FPSILCHLPSLHSLSLYNNSLNGSLSADDFDTCHNLISLDLSENLLVGSIPKSLPFNLPNLKFLEISGNNLSDTIPSSFG  160
EFRKLESLNLAGNFLSGTIPASLGNVTTLKELKLAYNLFSPSQIPSQLGNLTELQVLWLAGCNLVGPIPPSLSRLTSLVN  240
LDLTFNQLTGSIPSWITQLKTVEQIELFNNSFSGELPESMGNMTTLKRFDASMNKLTGKIPDNLNLLNLESLNLFENMLE  320
GPLPESITRSKTLSELKLFNNRLTGVLPSQLGANSPLQYVDLSYNRFSGEIPANVCGEGKLEYLILIDNSFSGEISNNLG  400
KCKSLTRVRLSNNKLSGQIPHGFWGLPRLSLLELSDNSFTGSIPKTIIGAKNLSNLRISKNRFSGSIPNEIGSLNGIIEI  480
SGAENDFSGEIPESLVKLKQLSRLDLSKNQLSGEIPRELRGWKNLNELNLANNHLSGEIPKEVGILPVLNYLDLSSNQFS  560
GEIPLELQNLKLNVLNLSYNHLSGKIPPLYANKIYAHDFIGNPGLCVDLDGLCRK
................................................................................   80
.................N...N...............................................N..........  160
........................N........................N..............................  240
.........................................N......................................  320
................................................................................  400
................................................................................  480
................................................................................  560
.......................................................                            640
```

B HSL2 extracellular domain

```
MLTNTNLFFFLSLLLLSCFLQVSSNGDAEILSRVKKTRLFDPDGNLQDWVITGDNRSPQNWTGITCHIRKGSSLAVTTID   80
LSGYNISGGFPYGFCRIRTLINITLSQNNINGTIDSAPLSLCSKLQNLILNQNNFSGKLPEFSPEFRKLRVLELESNLFT  160
GEIPQSYGRLTALQVLNLNGNPLSGIVPAFLGYLTELTRLDLAYISFDPSPIPSTLGNLSNLTDLRLTHSNLVGEIPDSI  240
MNLVLLENLDLAMNSLTGEIPESIGRLESVYQIELYDNRLSGKLPESIGNLTELRNFDVSQNNLTGELPEKIAALQLISF  320
NLNDNFFTGGLPDVVALNPNLVEFKIFNNSFTGTLPRNLGKFSEISEFDVSTNRFSGELPPYLCYRRKLQKIITFSNQLS  400
GEIPESYGDCHSLNYIRMADNKLSGEVPARFWELPLTRLELANNNQLQGSIPPSISKARHLSQLEISANNFSGVIPVKLC  480
DLRDLRVIDLSRNSFLGSIPSCINKLKNLERVEMQENMLDGEIPSSVSSCTELTELNLSNNRLRGGIPPELGDLPVLNYL  560
DLSNNQLTGEIPAELLRLKLNQFNVSDNKLYGKIPSGFQQDIFRPSFLGNPNLCAPNLDPIRPCRSKRET
...........................................................N....................   80
....N................N........N.................................................  160
.........................................................N..N...................  240
.................................................N............N.................  320
................................................................................  400
.....................................................................N..........  480
........................................................N.......................  560
......................................................................             640
```

Supplementary Figure 7: Predicted number and location of N-Glycans in HAE and HSL2 extracellular domains

Predictive software used to locate Asn-Xaa-Ser/Thr sequons present in HAE and HSL2 extracellular domains. A) Six of the nine sequons in the extracellular domain of HAE are expected to be glycosylated and B) 10 of 14 sequons in the extracellular domain of HSL2 are expected to be glycosylated.

Supplementary Figure 8:

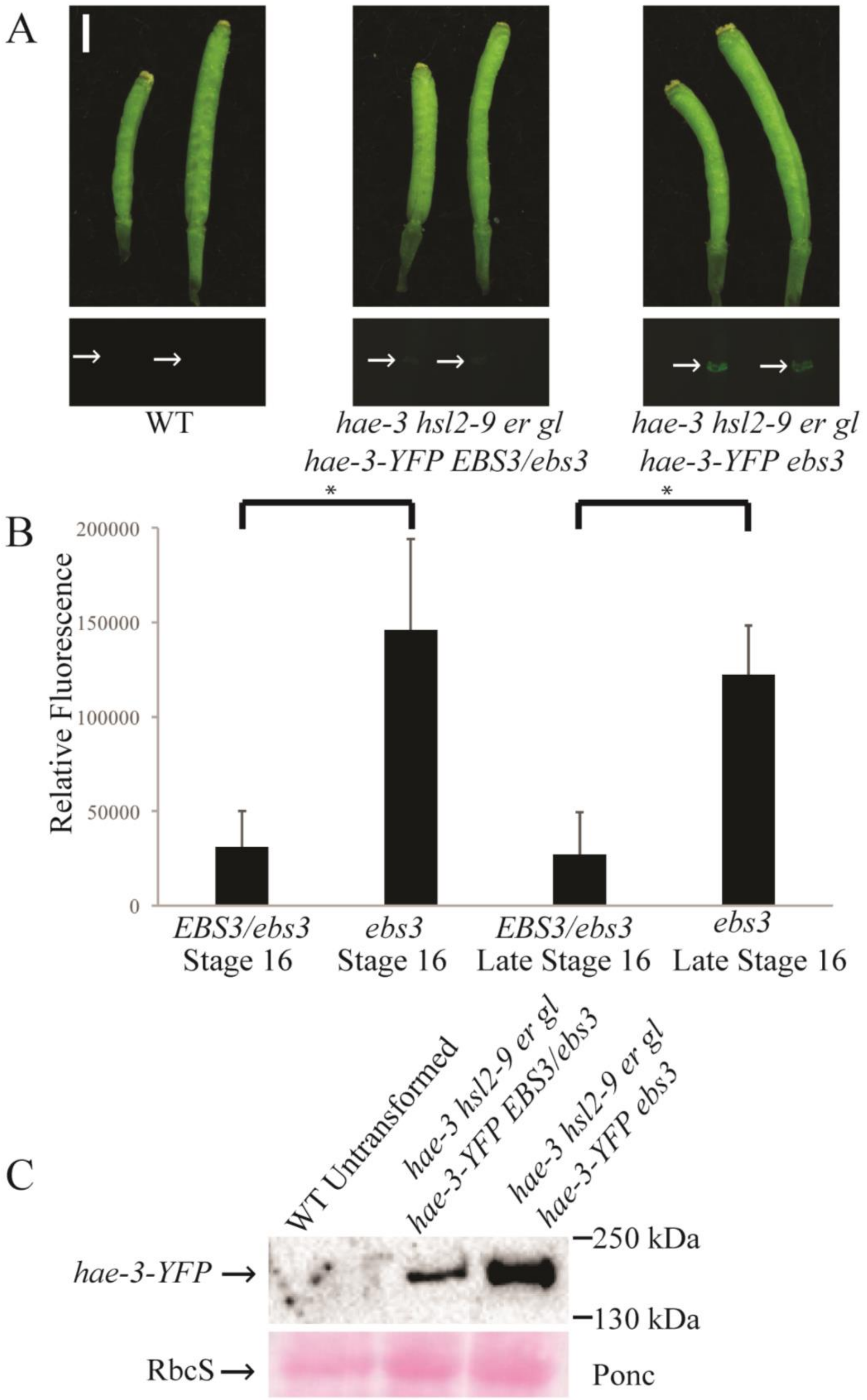

Supplementary Figure 8: Levels of hae-3-YFP receptor are higher in the *ebs3* mutant

A) Photographs (white light and YFP fluorescence) of stage 16 and late stage 16 siliques of wild type, *hae-3 hsl2-9 er gl hae-3-YFP EBS3/ebs3*, and *hae-3 hsl2-9 er gl hae-3-YFP ebs3*. Floral organs forcibly removed to expose abscission zones for all siliques. B) Quantification of YFP signal. Significant differences between EBS3/*ebs3* and *ebs3* for both stage 16 and late stage 16 siliques determined by Student's t-test (p-value<0.05, St. Dev. error bars shown). C) Western blot with anti-GFP antibody showing higher detection of hae-3-YFP in *ebs3* mutant than the *EBS3*/*ebs3* mutant. Bands detected are close to the expected size of hae-3-YFP (estimated at 156kDa). Ponceau stain (bottom) for loading control.
